# Simulation test study on filling flow law of gangue slurry in goaf

**DOI:** 10.1038/s41598-023-45596-0

**Published:** 2023-11-05

**Authors:** Zhanshan Shi, Hanwei Zhao, Bing Liang, Weiji Sun, Jian Wang, Shengjie Fang

**Affiliations:** 1 School of Mining, Liaoning Technical University, Fuxin, 123000 China; 2 School of Mechanics and Engineering, Liaoning Technical University, Fuxin, 123000 China; 3 Liaoning Academy of Mineral Resources Development and Utilization Technical and Equipment Research Institute, Liaoning Technical University, Fuxin, 123000 China

**Keywords:** Energy science and technology, Engineering

## Abstract

The disposal and utilization of solid waste of coal gangue is one of the main problems in coal mining in China. Injecting coal gangue into goaf in the form of slurry can effectively solve the problems of ground stacking and environmental pollution prevention. In order to obtain the flow law of gangue slurry in the void of the accumulated rock in the goaf, a visualization simulation test device for gangue slurry permeation grouting in the goaf was independently designed. The flow and diffusion characteristics, flow and diffusion velocity changes, void pressure changes, and viscosity changes of three mass concentrations (76%, 78%, 80%) of gangue slurry in the void between caved rock blocks in goaf were studied by visual grouting simulation test. The results show that: (1) The seepage process of gangue slurry in the goaf simulation test is divided into three diffusion forms, namely radial diffusion, axial diffusion, and bidirectional diffusion. The three diffusion forms are interrelated and inseparable. (2) The initial flow velocity of the slurry with different concentrations is different under the same permeation grouting pressure, and the higher the slurry concentration, the smaller the initial flow velocity of the slurry. The velocity of the slurry has a nonlinear relationship with the diffusion distance of the slurry. (3) With the permeation and diffusion of slurry, pressure sensors at different positions are subjected to pressure from bottom to top and enter the pressure boost stage, gradually forming stress peaks. When the slurry exceeds the position of the pressure sensor, the pressure on the pressure sensor is weakened and begins to enter the pressure relief stage, and the stress decline trend gradually becomes gentle with time. (4) The water loss effect occurs during slurry flow interaction with rock mass, resulting in slurry viscosity increasing. The viscosity of the slurry affects the difference in the amount of viscosity change. The research results can provide a certain theoretical basis for the goaf gangue slurry filling project.

## Introduction

In the process of coal resource mining and consumption utilization, a large amount of coal gangue solid waste is accompanied^1,2^. At present, the western mining area has become the main production capacity base of coal in China, and the number of 10 million tons of mines is constantly increasing. The discharge of gangue generated by them is also increasing year by year^3–5^. The total amount of coal gangue in China has reached more than 7 billion tons. Except for a small amount of comprehensive utilization^6^, the rest of the coal gangue is mainly processed by stacking on the ground, occupying a large amount of land resources and polluting the air, groundwater, and soil, resulting in a series of ecological and environmental problems^7–9^. Long-term theoretical research and practice have proved that using underground filling technology to treat coal gangue is the most reliable, thorough, and green disposal method with the lowest supervision cost at present, which not only solves the problem of coal gangue accumulation but also reduces and alleviates the surface settlement to a certain extent^10,11^. At present, China’s abandoned goaf is rich in space resources^12^. A certain concentration of gangue slurry can be prepared from coal gangue, fly ash, and water through the ground pulping system, and the gangue slurry can be filled in the caving accumulated rock mass in the goaf, providing a green disposal approach to solve the problem of ground stacking of coal gangue.

The gangue slurry filling in goaf is a new coal green mining technology developed from the traditional solid filling^13–15^, paste filling^16–18^, and overburden separated layer grouting filling^19–21^. At present, the research on gangue slurry filling is in the initial stage, and its industrialization test, theoretical basis, and technical system are still being gradually established. The existing theory of gangue slurry diffusion can not be well applied to the engineering practice of gangue slurry injection in goaf. Therefore, it is of great significance to study the flow diffusion law of gangue slurry for the development of grouting theory and engineering.

Sun et al.^22^ studied the effects of gangue mass fraction, particle gradation and standing time on the rheological properties of gangue filling slurry, and confirmed that the characteristics of coal gangue slurry belong to Bingham fluid. Jin et al.^23,24^ analyzed the particle size distribution of coal gangue-fly ash mixture under different mixing ratios, and studied the effects of gradation and concentration on the rheological parameters of coal gangue-fly ash slurry. Wang et al.^25^ obtained the spatial distribution expression of viscosity decay rate considering the influence of water-cement ratio through a one-dimensional visual grouting test, and derived the Bingham fluid column permeability grouting theoretical formula considering the spatial decay of viscosity. Yang et al.^26^ proposed a Bingham fluid column permeation and diffusion mechanism considering the effect of grout on groundwater displacement. Wang et al.^27^ derived the conventional permeation and diffusion mechanism of Bingham fluid in porous media based on the equation of seepage motion. Self-gravity is one of the key parameters to characterize the permeation and diffusion behavior of grout. Fu et al.^28^ proposed a mathematical model of permeation grouting considering the effect of grout’s self-weight. Zhu et al.^29^ used the self-designed visual test platform for slurry diffusion in goaf to monitor the flow diffusion range of coal gangue slurry in the caving zone of goaf by using the active heating fiber method (AHFO). Shi et al.^30,31^ studied the diffusion behavior of grouting slurry under different conditions through an experimental system simulating the development of cracks in overlying strata in the goaf. By establishing numerical models, Wang et al.^32^ studied the diffusion and rock displacement laws of fluidized gangue with different grouting speeds, pore ratios, and gangue particle sizes. Wang et al.^33^ studied the grouting seepage of micro-cracks under different grouting pressure and crack opening conditions through numerical experiments, and obtained the change law of the spatial distribution of crack opening and the change law of slurry seepage distance under grouting pressure. Wang et al.^34,35^ established a single crack grouting model considering fluid-structure coupling, and carried out numerical simulation research on the multi-physical coupling mechanism. Xie et al.^36^ used COMSOL Multiphysics finite element analysis software to build a numerical model and solve the grouting part of the advanced grouting anchor cable. Some scholars^37–41^ have also done a lot of research on the influence of rock pore structure and permeability on fluid in theoretical analysis and numerical simulation.

To sum up, scholars at home and abroad have studied the rheological parameters and characteristics of gangue slurry, the mechanism of infiltration and diffusion, and the diffusion behavior under different conditions, but there are few studies on the flow rule of gangue slurry in the accumulated rock mass of goaf, the coupling effect of slurry and rock, and the effect of water loss. In view of the above problems, this paper carried out a visual simulation study on the flow and diffusion process of gangue slurry in accumulated rock during the injection process of gangue slurry in goaf. The seepage diffusion characteristics, the variation of slurry flow diffusion, the change of pore pressure, and the viscosity of gangue slurry in the voids between the caving rocks in the goaf were obtained with three mass concentrations (76%, 78%, and 80%).

## Selection and ratio of basic parameters of gangue slurry materials

The main components of the gangue slurry used in the test are gangue powder, fly ash, and water. The injection time is short, and the viscosity timeliness will not occur in a certain period of time^22,42^. The main viscosity change of gangue slurry is only related to the injected medium and the seepage channel. The experimental design slurry concentration was 76%, 78%, and 80%.

**Figure1:**
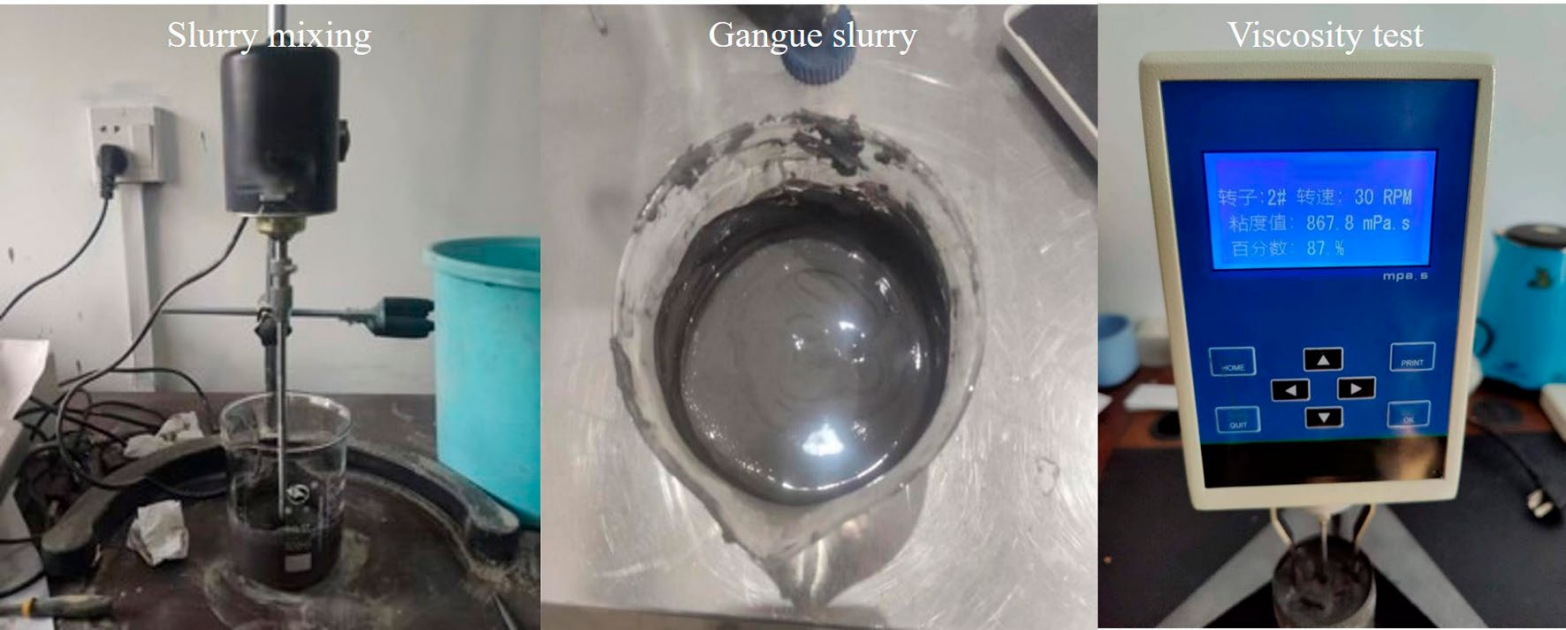
Viscosity measurement.

**Figure2:**
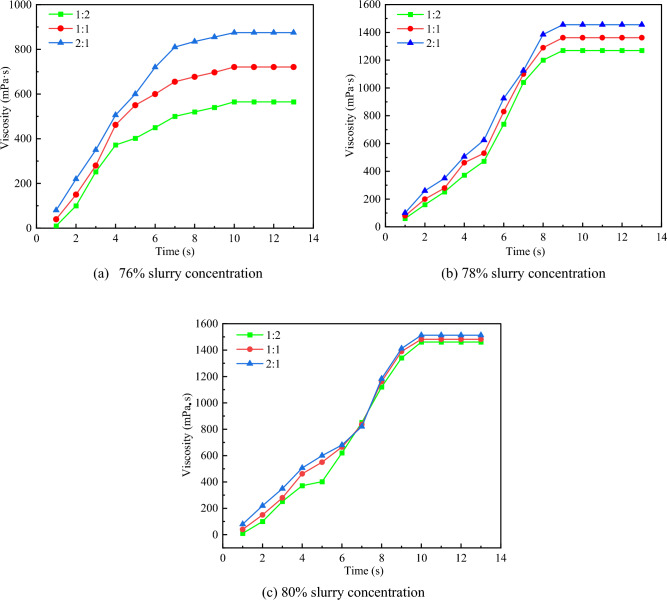
Slurry viscosity measurement.

Through the viscosity measurement test (Fig. 1), the influence of different proportions of gangue powder and fly ash on slurry viscosity under the conditions of three slurry concentrations was tested. The ratio of gangue powder to fly ash is 1:2, 1:1, 2:1, and the change of slurry viscosity under the three ratio conditions is measured respectively. The NDJ-9s digital viscometer was used to measure the viscosity of slurry, and the viscosity values of 76%, 78%, and 80% were recorded over time. Based on the measurement data, the viscosity curve of different ratios of gangue powder to fly ash over time is drawn under the condition of different slurry concentrations. The viscosity measurement results are shown in Fig. 2.

It can be seen from Fig. 2 that in the viscosity measurement process, the viscosity of slurry of different concentrations increases with the increase of measurement time, and the slurry viscosity remains unchanged when the maximum slurry viscosity is reached. Under different proportions of gangue powder and fly ash, the viscosity of slurry with lower concentration is significantly affected, and the larger the proportion of fly ash, the smaller the viscosity of slurry. While for gangue slurry with higher concentration, the fly ash has little effect on slurry viscosity. Due to the increasing demand for fly ash and the high transportation cost, the efficiency of filling mining is affected. At the same time, in order to solve the problem of gangue stacking and increase the consumption of gangue, a large proportion of gangue powder is needed. Therefore, the choice of gangue powder and fly ash of 2:1 for the test. The mixture of gangue slurry is shown in Table 1.

**Table 1:** The ratio of gangue slurry.

Slurry concentration (%)	Quality of gangue powder /kg	Fly ash quality /kg	Quality of water /kg	Initial viscosity /mps·s
76	20.2	10.2	9.6	852
78	20.8	10.4	8.8	1270
80	21	11	8	1461

## Simulation test of gangue grouting in goaf

### Constitution of the test device

**Figure 3:**
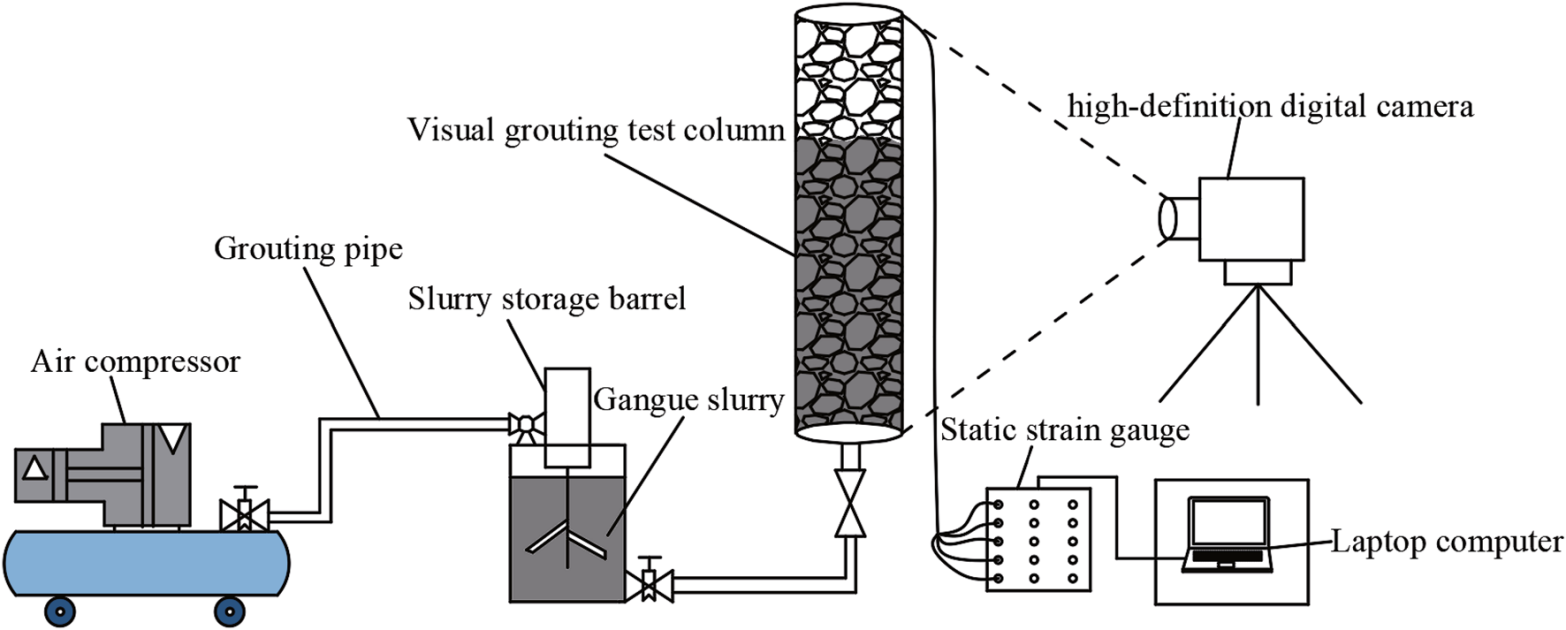
Test device diagram.

**Figure 4:**
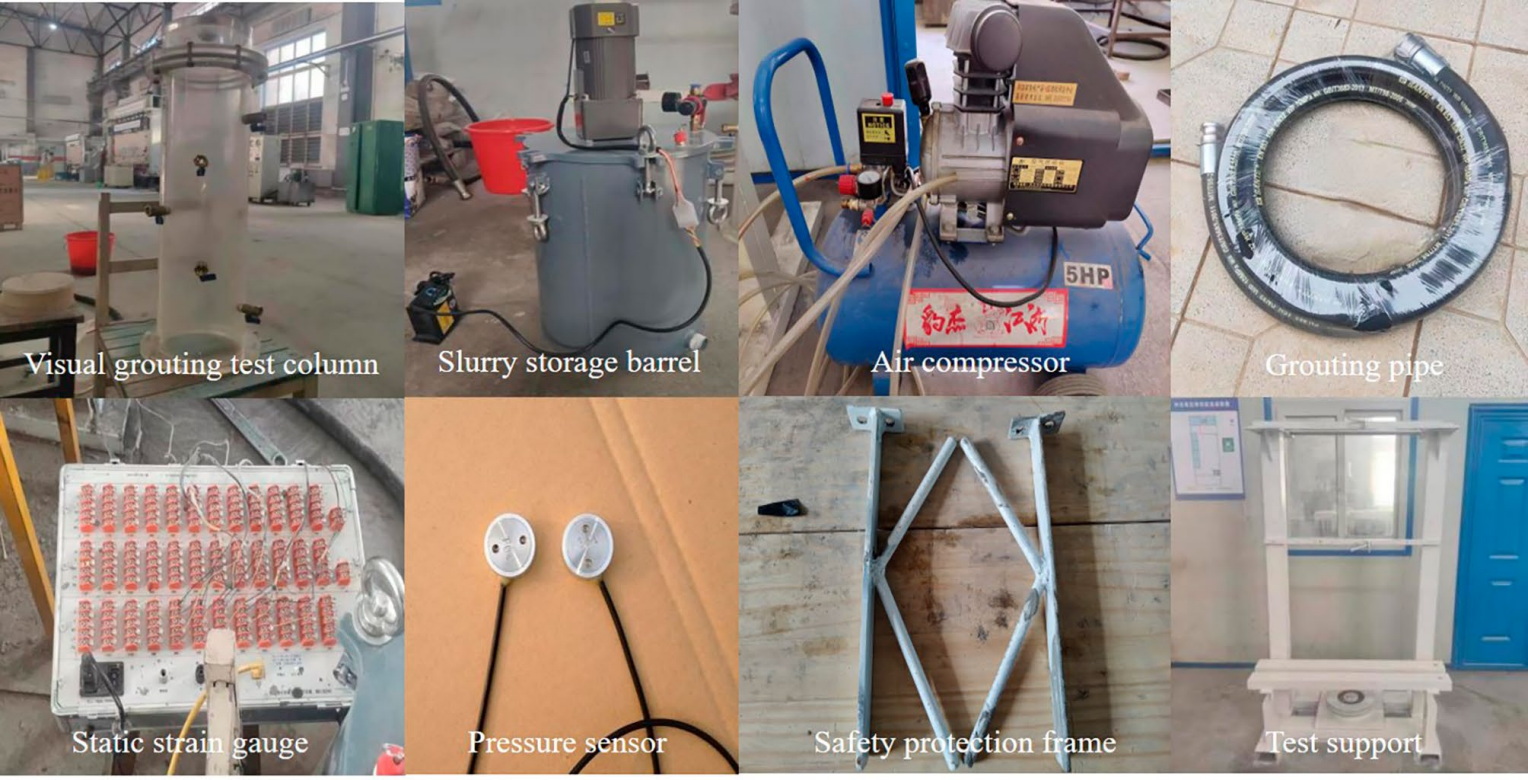
Physical picture of device components.

The physical simulation test equipment of gangue slurry infiltration in goaf includes grouting system, goaf simulation system, data monitoring and collection system, and safety protection system. The schematic diagram of the test device is shown in Fig. 3, and the physical diagram of each component is shown in Fig. 4.

The grouting system consists of an air compressor, a slurry storage barrel, and a grouting pipe. The maximum pressure of the slurry storage barrel is 0.8 MPa. The goaf simulation system includes visual grouting test column and simulated accumulated rock mass. The grouting test column is made of transparent acrylic plate. The hollow column has a wall thickness of 10 mm, a height of 700 mm, an inner diameter of 200 mm, and an outer diameter of 220 mm. Each end is connected with a flange. The visual grouting test column is provided with a fixed blocking stone device at the top, which only allows the slurry to pass through and prevents the whole movement of the injected medium during the grouting process. The data monitoring and acquisition system mainly includes static strain gauge, pressure sensor, laptop computer, NDJ-9s rotary viscometer, and high-definition digital camera. The safety protection system is composed of test support and safety protection frame.

### Test materials and voidage measurement 

#### Test materials

**Figure 5:**
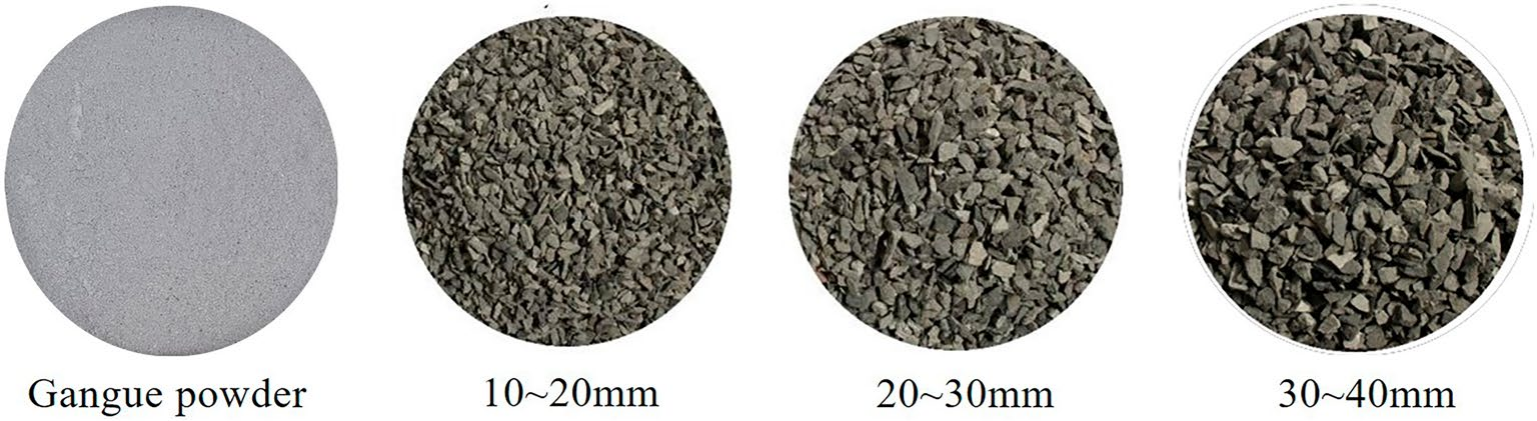
Coal gangue material.

Gangue blocks were used as the injected medium. The gangue samples used in the simulation test were taken from Fuxin Mining Group. The gangue is crushed and screened by the jaw crusher, as shown in Fig. 5. The remaining particle size was crushed and screened for gangue powder and used for the preparation of gangue slurry. The simulated rock blocks are dried before each grouting test to ensure that the rock blocks are dry and put into the visual grouting test column. The gradation of simulated accumulations is shown in Table 2.

**Table 2:** Simulated accumulative rock mass classification.

Particle size of gangue/mm	Proportion /%	Quality /kg
10–20	22.14	6.20
20–30	33.57	9.40
30–40	44.29	12.40

#### Measurement of voidage of simulated accumulated rock mass

Before the test, the gangue blocks with three particle sizes of 10–20 mm, 20–30 mm, and 30–40 mm were dried, and the visual grouting test column was sealed, its internal volume was measured, and the dried gangue blocks were put into the visual grouting test column in proportion until the pile was flush with the pipe mouth. Take a bucket filled with water and measure the mass $m_{1}$, pour the water in the bucket into a visual grouting test column filled with gangue until the liquid level is flush with the pipe mouth, then measure the mass $m_{2}$ of the remaining water and the bucket. The parameters are shown in Table 3, which is calculated as 43.16% by substituting them into the calculation formula 1.

1 $$V_{c} = \frac{{(m_{1} - m_{2} )/\rho }}{V} \times 100\%$$

where $V_{{\text{c}}}$ is the void ratio of simulated accumulation body; $m_{1}$ is the mass of water and bucket; $m_{2}$ is the mass of the remaining water and the bucket; $\rho$ is the density of water; $V$ is the volume of visual grouting test column.

**Table 3:** Parameters.

$V$/L	$m_{1}$/kg	$m_{2}$/kg	$m_{1} - m_{2}$/kg	$\rho$/kg·m^3^
21.5	15.0	5.72	9.28	1.0

### Experimental design

#### Test scheme

In order to facilitate the analysis of the law of gangue slurry under different grouting parameters, three kinds of gangue slurry with mass concentration (76%, 78%, 80%) were selected respectively, and the simulation test of gangue slurry grouting in goaf was carried out under the conditions of the same deposit void ratio and different grouting pressure. A total of 9 groups of simulation tests were designed, as shown in Table 4.

**Table 4:** Experimental design scheme.

Number	Slurry concentration (%)	Grouting pressure /MPa	Voidage (%)
1	76	0.2	43.16
2	76	0.3	43.16
3	76	0.4	43.16
4	78	0.2	43.16
5	78	0.3	43.16
6	78	0.4	43.16
7	80	0.2	43.16
8	80	0.3	43.16
9	80	0.4	43.16

#### Test process

1. Preparation and screening of gangue blocks with different particle sizes and grinding gangue powder.
2. Assemble test equipment. Each system device is connected, the simulated accumulation of rock blocks is arranged in the visual test column, and the pressure sensor is arranged in the center of the shaft. A pressure sensor is installed every 15 cm from the bottom to the top, the initial layout height is 5 cm, and the pressure sensor numbers are 1, 2, 3, 4, and 5 from the bottom to the top. Pressure sensors with the same number were placed at the same height for each group of tests.
3. Preparation of gangue slurry. Mix gangue powder, fly ash, and water in proportion and use an industrial electric mixer to stir well.
4. Visual grouting simulation test. The air compressor is used to apply constant pressure to the slurry storage barrel to start grouting, and the grouting stops when the gangue slurry is gushing out in large quantities at the top of the visual grouting test column. Static strain gauge was used to monitor and collect the dynamic change data of void pressure, and high-definition digital camera was used to record the whole process of seepage and diffusion of gangue slurry.
5. Test the change of slurry viscosity. After the end of grouting, the gangue slurry filling body was taken out, the slurry penetration filling result was recorded, and the slurry at different diffusion heights was extracted to test the slurry viscosity.
6. Save the recorded data and disassemble the cleaning test device. Repeat the above steps for the grouting test of the remaining groups.

#### Measurement of test parameters

**Figure 6:**
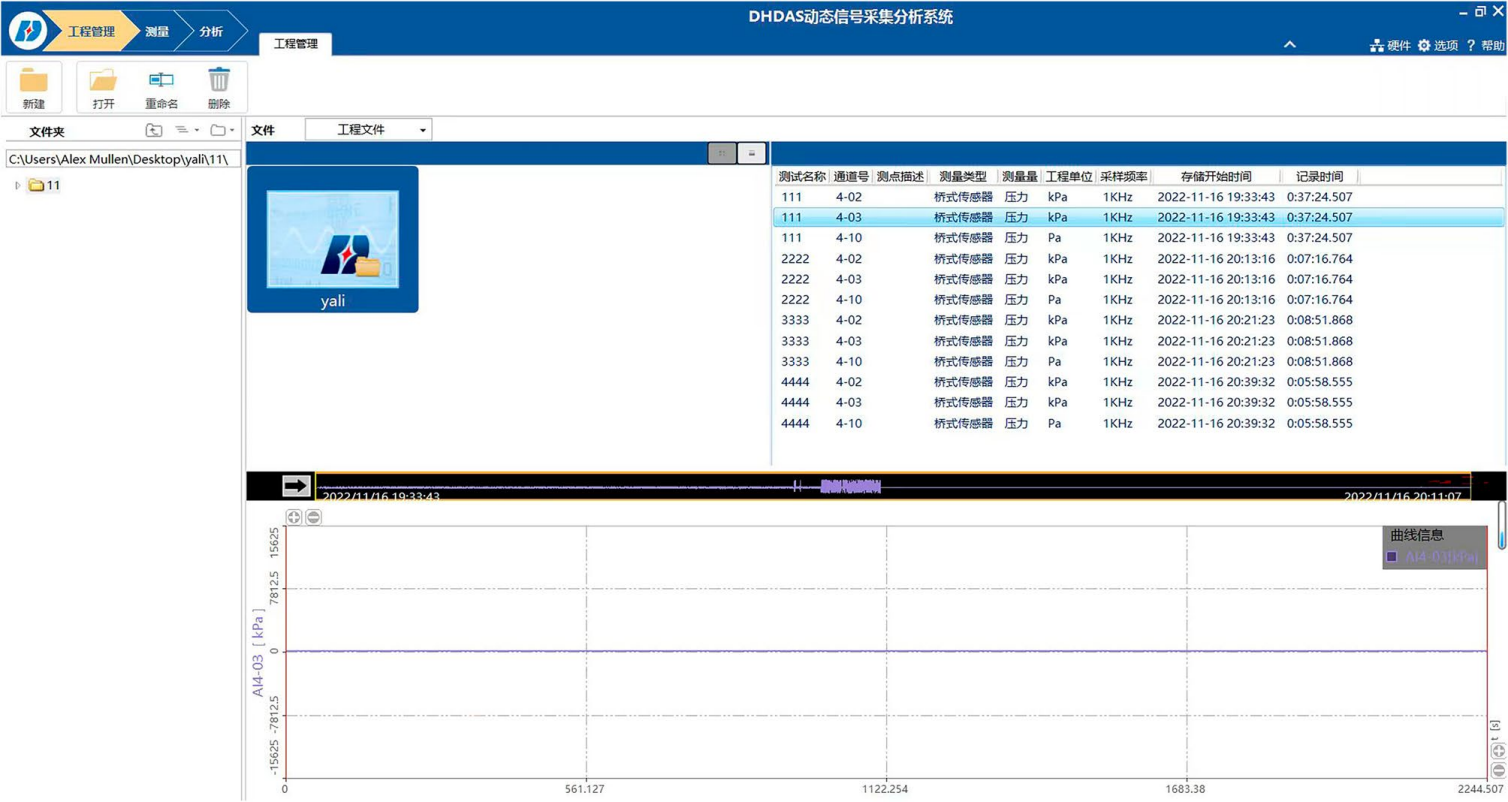
Data acquisition and analysis system.

**Figure 7:**
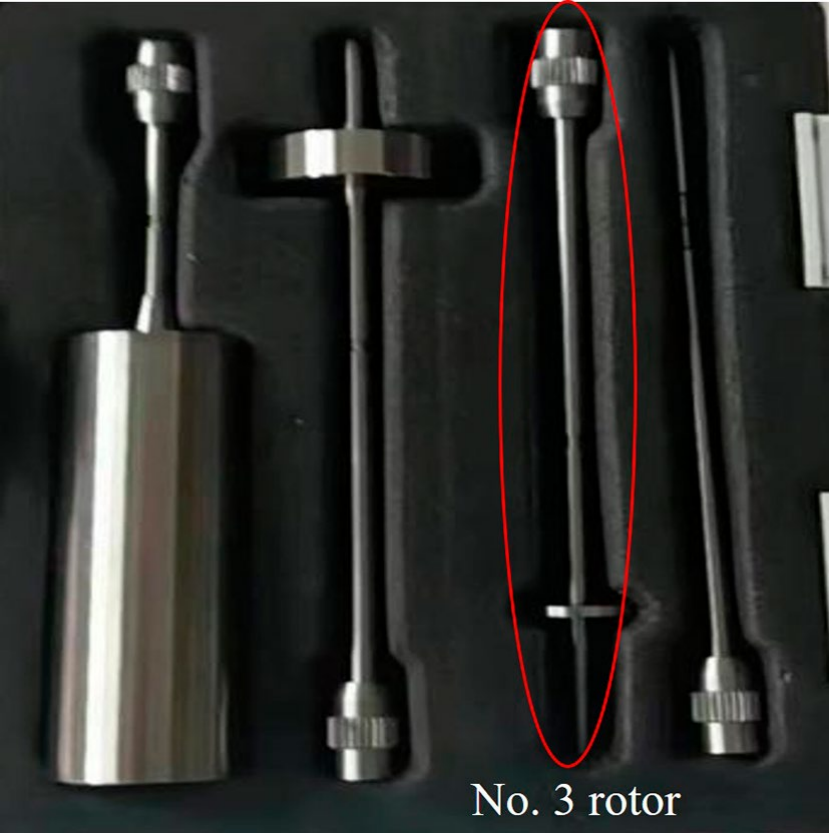
Rotor.

- (1) Slurry velocity measurement.
  Due to the visualization of the grouting simulation test system, high-definition digital camera was selected to monitor and record, and the data was imported into the processing software to solve and analyze the flow rate of the gangue slurry liquid level rising.
- (2) Measurement of void dynamic pressure change.
  In order to monitor the dynamic pressure change between the simulated stacked rock blocks, static strain gauge and pressure sensor were used for monitoring, and the acquisition frequency was 0.2s. The connection mode of the measuring bridge is full bridge. The dynamic data acquisition and analysis system (Fig. 6) matching DHDAS software is used to set the parameters of pressure sensors with different numbers. After balancing and zeroing the measuring point, the pressure change data is collected.
- (3) Measurement of slurry viscosity change.
  Pour the slurry extracted from different diffusion heights into 250mL beakers and stir well. The No. 3 rotor of NDJ-9s rotary viscometer (Fig. 7) was selected and the speed was set at 30r/min. The viscosity of slurry was measured and recorded successively.

## Analysis of test results

### Seepage process and characteristics of slurry

**Figure 8:**
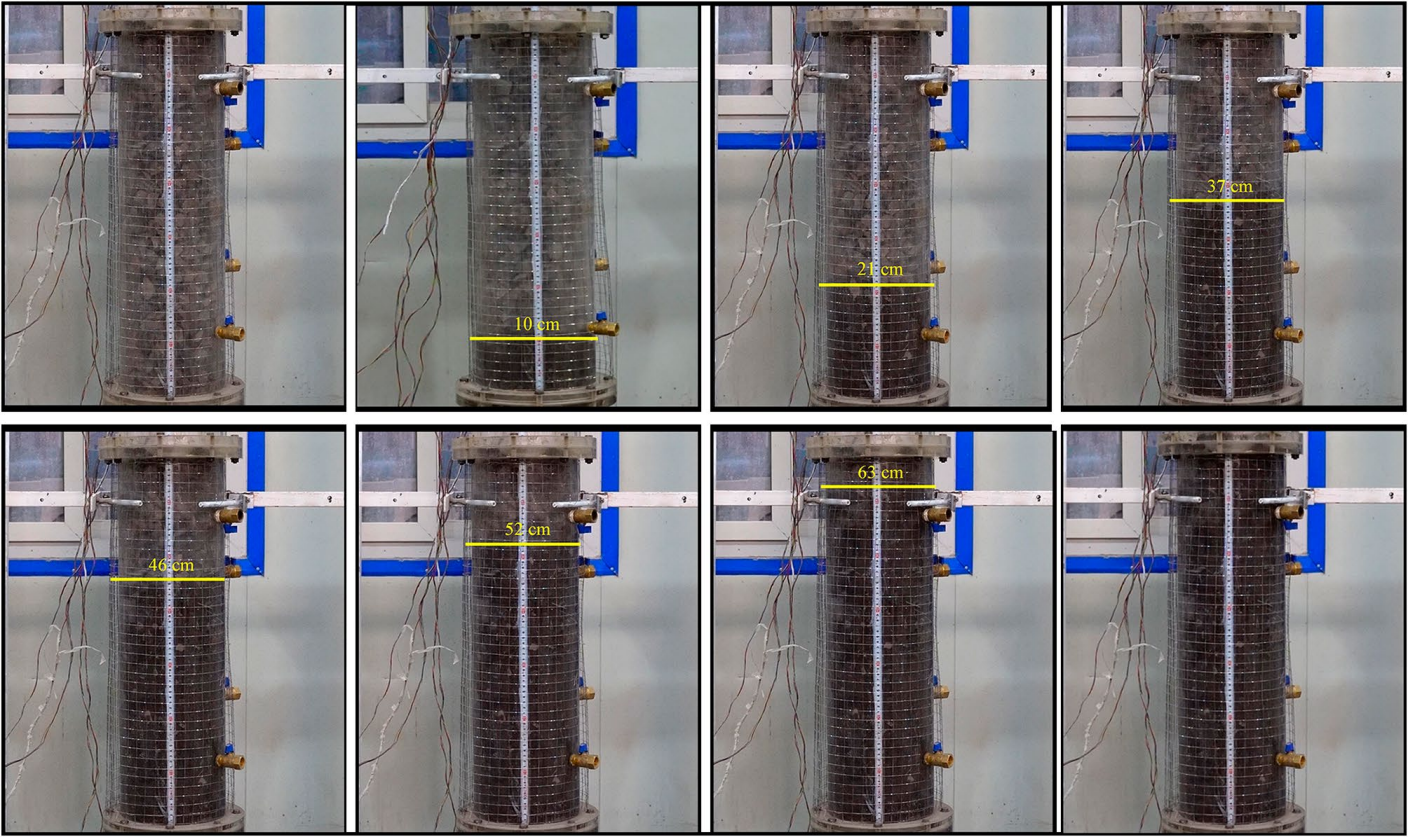
Seepage and diffusion process of slurry.

**Figure 9:**
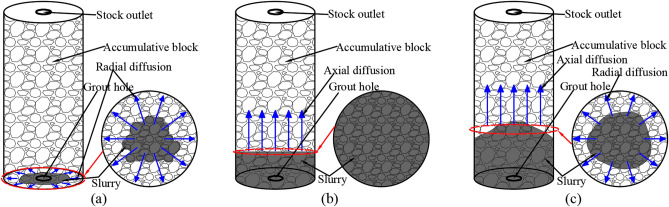
Seepage characteristics.

According to the flow law of gangue slurry in the visual grouting test column in the grouting test process (Fig. 8), it can be divided into three stages, which are interrelated and inseparable. When the gangue slurry is initially injected, the gangue slurry takes the grouting mouth as the circle center and diffuses around the void of the simulated rock block. This stage is called the radial diffusion stage^43^, as shown in Fig. 9a. When the gangue slurry reaches the boundary of the simulated rock void, the diffusion front of the slurry begins to find the dominant path, that is, it permeates and diffuses to the void channel with lower flow resistance, showing an uneven axial diffusion flow characteristic. This stage is called the axial diffusion stage, as shown in Fig. 9b. In the process of axial flow diffusion of gangue slurry, when the body weight of the slurry, the simulated self-gravity and void resistance of the upper part of the slurry are greater than the simulated lateral resistance of the void of the slurry in the process of radial diffusion of the slurry, the slurry will flow into the surrounding void and squeeze the rock block. There are both radial diffusion and axial diffusion flow characteristics, and this stage is called the bidirectional diffusion stage, as shown in Fig. 9c.

**Figure 10:**
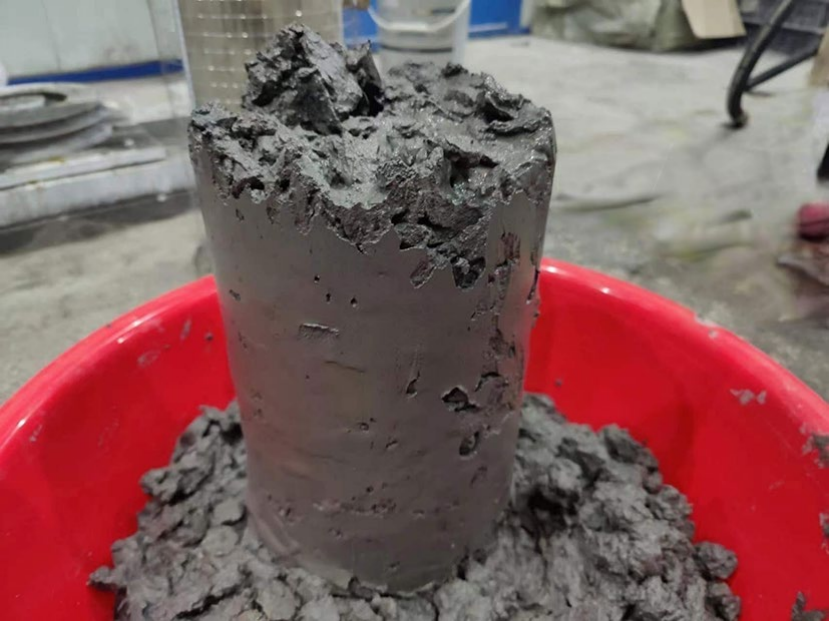
Grouting filling effect.

After the grouting test, the mixture of simulated accumulated rock and gangue slurry in the test column was taken out, and the slurry was evenly filled into the void of simulated accumulated rock mass to form uniform grouting plus solid, and the grouting and filling effect was good, as shown in Figure 10.

### Variation of slurry flow diffusion velocity

Under the same grouting pressure condition, the flow velocity changes of 76%, 78%, and 80% slurry with different concentrations are compared. The test results are shown in Table 5.

**Table 5:** Test results of slurry flow velocity change.

Number	Slurry concentration (%)	Grouting pressure /MPa	Slurry velocity /(cm/s)						
			10cm	20cm	30cm	40cm	50cm	60cm	70cm
1	76	0.2	15.30	12.50	9.65	7.10	5.67	4.74	3.67
2		0.3	20.00	15.00	11.80	9.50	8.30	6.67	5.30
3		0.4	23.33	18.76	15.20	11.8	9.76	8.50	6.90
4	78	0.2	7.80	5.70	4.90	4.00	3.64	3.33	3.08
5		0.3	11.07	8.25	6.67	5.56	4.95	4.58	4.25
6		0.4	15.00	11.20	9.41	8.20	6.95	6.32	5.95
7	80	0.2	2.90	2.45	1.80	1.36	0.97	0.65	0.50
8		0.3	4.20	3.60	3.05	2.40	1.80	1.45	1.10
9		0.4	5.20	4.50	3.90	3.40	2.90	2.30	1.75

**Figure 11:**
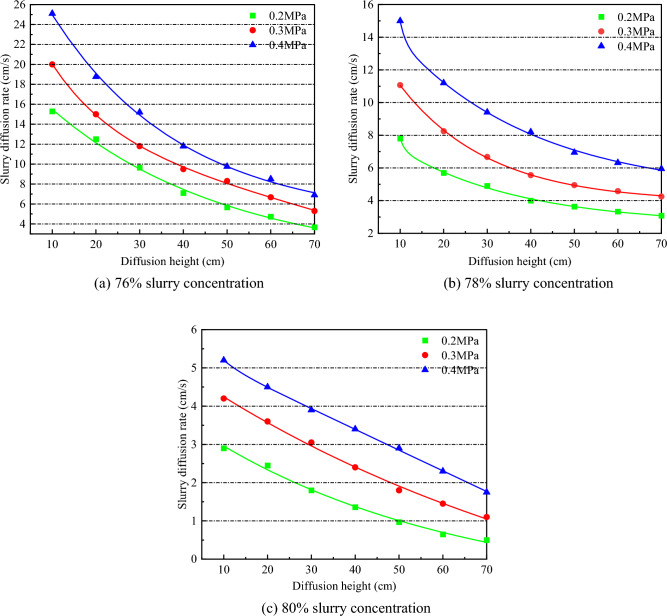
Change of slurry flow velocity under different grouting pressure.

**Figure 12:**
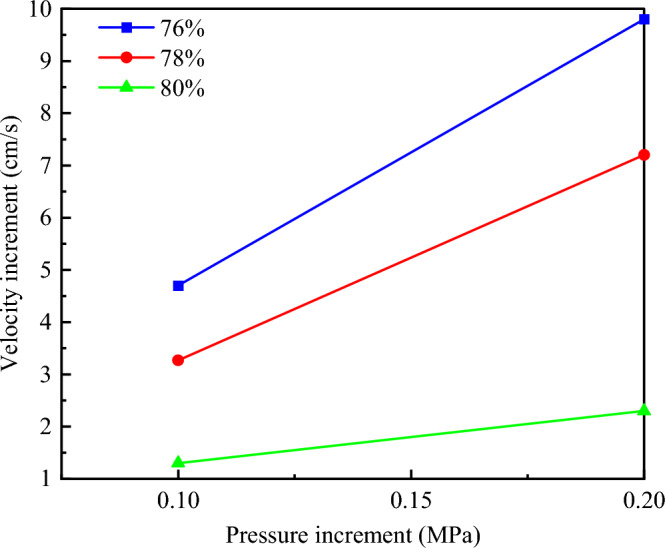
Relation between grouting velocity increase and grouting pressure increase.

It can be seen from the analysis of Fig. 11 that the flow velocity of slurry with different concentrations is different under the same grouting pressure, and the larger the slurry concentration, the smaller the slurry flow velocity. With the increase of grouting pressure, the flow rate of slurry with different concentrations increases. Taking the slurry flow velocity at the diffusion height of 10 cm as an example, the slurry flow velocity of 76% slurry at 0.2 MPa grouting pressure was 15.3 cm/s, and that at 0.3 MPa and 0.4 MPa grouting pressure was 20 cm/s and 25.1 cm/s, respectively, increasing by 30.72% and 64.05%. The slurry flow velocity of 78% slurry was 7.8 cm/s under 0.2 MPa grouting pressure, and 11.07 cm/s and 15 cm/s under 0.3 MPa and 0.4 MPa grouting pressure, increasing by 41.92% and 92.31%, respectively. The slurry flow velocity of 80% concentration slurry was 2.9 cm/s under 0.2 MPa grouting pressure, and 4.2 cm/s and 5.2 cm/s under 0.3 MPa and 0.4 MPa grouting pressure, which increased by 44.83% and 79.31%, respectively. There is a linear relationship between the growth rate of slurry flow velocity of the three concentrations and the growth rate of grouting pressure, as shown in Fig. 12. Moreover, under the condition of the same pressure increase, the larger the slurry concentration, the lower the growth rate of flow velocity.

There is a nonlinear relationship between slurry velocity and slurry diffusion distance, which indicates that the flow of gangue slurry in the accumulated rock mass is nonlinear, and slurry velocity decreases gradually with the increase of diffusion distance. This is because the voidage of the accumulated rock mass is a constant value. As the slurry permeates into the voidage of the accumulated rock mass, the voidage gradually decreases and the flow resistance gradually increases, resulting in the gradual attenuation of the slurry velocity, and the attenuation rate decreases with the increase of the diffusion distance. Under the condition of the same slurry concentration, the greater the grouting pressure, the faster the slurry velocity decay rate. Therefore, in the actual engineering grouting, it is necessary to design the viscosity parameters of the slurry to control the grouting efficiency and implementability.

### Change law of void pressure

**Figure 13:**
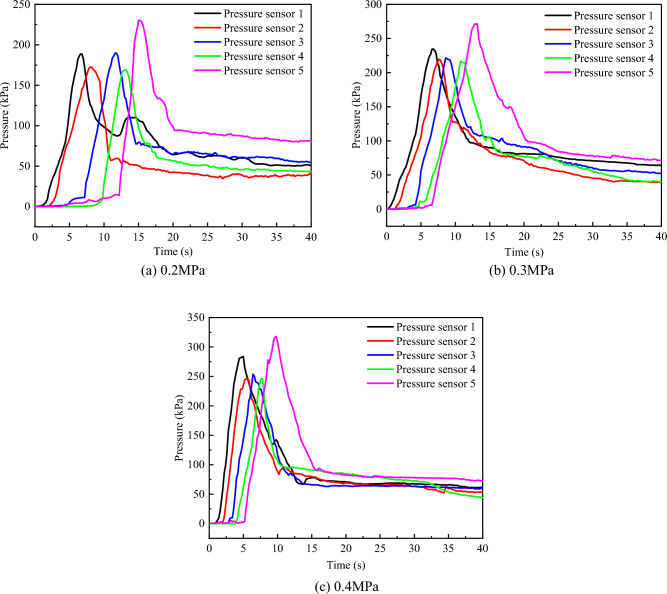
Interstitial pressure curve of accumulated rocks.

The 78% concentration gangue slurry was selected to compare the change rule of void pressure between the stacked rock blocks with the grouting time during the grouting process under the three grouting pressures of 0.2 MPa, 0.3 MPa, and 0.4 MPa. The dynamic change curve of its internal pressure during the grouting process is shown in Fig. 13.

The process of grouting test starts from the bottom up. In the process of seepage and diffusion of the slurry, upward thrust will be generated on the simulated formation inside the visual grouting test column. Therefore, a fixed hole plug net is set on the upper end of the test column to prevent the displacement and sliding of the simulated formation inside the test column. As the slurry gradually rises, the pressure sensors at different positions are subjected to pressure from bottom to top and gradually form peaks. When the slurry exceeds the position of the pressure sensor, the flowing slurry produces upward extrusion pressure on the accumulated rock above the pressure sensor, which causes the pressure on the pressure sensor to weaken and begin to enter the pressure relief stage, and the stress decreasing trend gradually becomes gentle.

According to the analysis of Fig. 13a, under the grouting pressure of 0.2MPa, the stress peak value formed by pressure sensor 5 is larger than that formed by pressure sensors 1, 2, 3, and 4. It can be seen from Fig. 13b,c that under the grouting pressure of 0.3MPa and 0.4MPa, the maximum stress peak of the pressure sensor is also generated at the top pressure sensor 5. The maximum stress peaks under the three grouting pressures are 230.18 kPa, 271.55 kPa, and 317.84 kPa, respectively. Compared with the three grouting pressures, with the increase of grouting pressure, the slurry diffusion speed also increases, the initial moment when the pressure sensor is affected by the slurry flow pressure is advanced, and the rise rate of the peak stress formed at each pressure sensor is faster. As the slurry is gradually filled into the accumulated rock blocks, the void ratio between the rock blocks gradually decreases, resulting in the higher the diffusion height, the greater the seepage and diffusion resistance of the slurry, so the maximum stress peak is formed at the top, and the greater the grouting pressure, the higher the stress peak.

As the accumulation rock block is compressed by the pressure of the gangue slurry, its porosity decreases, so that the permeability becomes lower, the flow resistance increases, and the flow rate decreases. The reduced flow rate of the gangue slurry prevents the internal pressure from rising, which in turn prevents the porosity in the simulated formation from being further compressed. Therefore, the observed peak pressure decreases from the bottom to the top, and the corresponding slurry velocity decreases rapidly at first and then gently.

### Change rule of slurry viscosity

**Figure 14:**
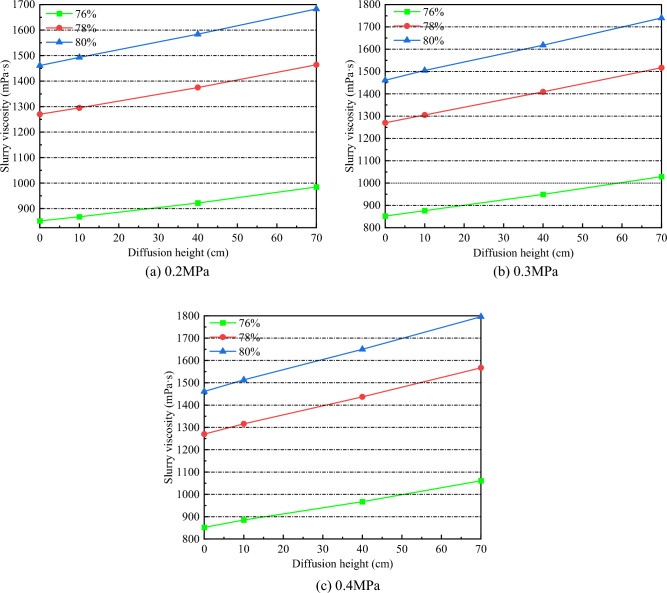
Slurry viscosity change.

It can be seen from the analysis of Fig. 14 that at the same diffusion height, the viscosity of gangue slurry with the same concentration increases with the increase of grouting pressure, and the higher the grouting pressure, the greater the change of slurry viscosity. Under different grouting pressures, slurry viscosity increases steadily with the increase of diffusion height. This is due to the water absorption of the rock mass in the goaf. In the process of simulating the flow of the rock mass in the goaf, the interaction between the rock mass and the slurry produces the seepage coupling phenomenon, resulting in the water loss effect of the slurry^44^, which results in the viscosity of the slurry increasing.

As can be seen from Table 6, under the grouting pressure of 0.2 MPa, the final viscosity of 76% concentration slurry increased by 15.61% compared with the initial viscosity value, and the 78% concentration slurry and 80% concentration slurry increased by 15.27% and 15.2% respectively. Under the grouting pressure of 0.3 MPa, the final viscosity of 76% concentration slurry increased by 20.77% compared with the initial viscosity, and the 78% concentration slurry and 80% concentration slurry increased by 19.45% and 19.10%, respectively. Under 0.4MPa grouting pressure, the final viscosity of 76% concentration slurry increased by 24.53% compared with the initial viscosity value, and the 78% concentration slurry and 80% concentration slurry increased by 23.39% and 22.93%, respectively. It can be seen that with the increase of grouting pressure, the difference between the final value of slurry viscosity and the initial value of slurry viscosity at the three concentrations of 76%, 78%, and 80% shows an upward trend. It shows that the greater the grouting pressure, the fuller the slurry rock coupling and the more obvious the slurry water loss effect, resulting in greater changes in slurry viscosity.

**Table 6:** Difference between initial viscosity and final viscosity.

Slurry concentration (%)	Grouting pressure /MPa	Initial viscosity /mPa·s	Final viscosity /mPa·s	Difference value /%
76	0.2	852	985	15.61
	0.3		1029	20.77
	0.4		1061	24.53
78	0.2	1270	1464	15.27
	0.3		1517	19.45
	0.4		1567	23.39
80	0.2	1461	1683	15.20
	0.3		1740	19.10
	0.4		1796	22.93

**Figure 15:**
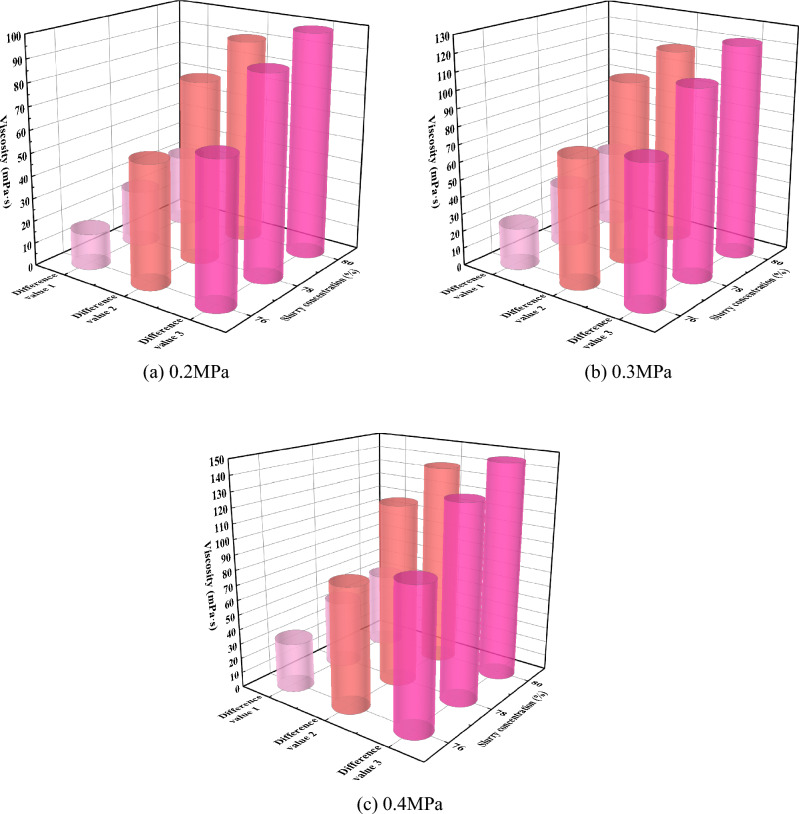
Three kinds of difference values change under different grouting pressure.

In order to further analyze the influence of slurry concentration on the change of slurry viscosity during diffusion, the difference values of three kinds of viscosity were obtained, as shown in Table 7. As can be seen from Fig. 15, under the same grouting pressure, the higher the slurry concentration, the greater the equal difference. For the same concentration of slurry, the three differences (differences 1, 2, 3) increased with the increase of the grouting pressure. Under the three grouting pressures, the higher the slurry concentration, the greater the change of the difference value, and the change of the difference value 3 at the same height of the slurry seepage diffusion is greater than that of the difference value 2. This indicates that when the slurry is diffused to the same height, the viscosity change of low-concentration slurry is smaller than that of high-concentration slurry, and the difference in viscosity change tends to rise with the increase of diffusion height.

**Table 7:** Three viscosity differences.

Grouting pressure /MPa	Slurry concentration (%)	Viscosity difference1 /mPa·s	Viscosity difference2 /mPa·s	Viscosity difference3 /mPa·s
0.2	76	16	54	63
	78	25	80	89
	80	32	91	99
0.3	76	24	73	80
	78	35	104	108
	80	44	113	122
0.4	76	33	82	94
	78	46	121	130
	80	52	137	146

Viscosity difference 1 is the difference between the viscosity at 10 cm of slurry diffusion and the initial viscosity; viscosity difference 2 is the viscosity difference between the diffusion of 10 cm and 40 cm; viscosity difference 3 is the viscosity difference between 40 and 70 cm.

## Conclusions

In this study, the self-designed visual grouting simulation test device was used to carry out the simulation test study on the flow law of gangue slurry filling in goaf. The seepage and diffusion characteristics of gangue slurry in simulated rock void in goaf were studied, and the changes of flow diffusion velocity, void pressure, and viscosity of gangue slurry in the seepage process were analyzed, which made an original contribution to the theoretical research of gangue slurry filling engineering in goaf. Due to the limitation of the test equipment, the mechanism of slurry coupling effect and water loss effect cannot be further studied from the microscopic perspective. In the future, CT scanning device can be used to analyze the flow and diffusion law of slurry in the void more comprehensively, study the influence of different rock physical and mechanical characteristics on the flow and diffusion of slurry, and the development and change of rock fractures during grouting process. The main conclusions are as follows:

1. The flow of gangue slurry in simulated rock voids in goaf can be divided into three diffusion forms, respectively radial diffusion, axial diffusion, and bidirectional diffusion. The three diffusion forms are interrelated and inseparable. In the flow diffusion process, slurry preferentially looks for the dominant path, that is, the void channel with less flow resistance.
2. Under the same grouting pressure, the initial flow velocity of slurry with different concentrations is different, and the higher the slurry concentration, the smaller the initial flow velocity of the slurry. With the increase of grouting pressure, the initial flow velocity of slurry with different concentrations increases accordingly. There is a nonlinear relationship between the velocity of slurry and the diffusion distance, and the velocity of slurry decreases with the increase of the diffusion distance. Under the condition of the same slurry concentration, the greater the grouting pressure, the faster the slurry velocity decay rate.
3. With the infiltration and diffusion of slurry, pressure sensors at different positions are subjected to pressure successively from bottom to top to enter the boost phase, and gradually form a stress peak. The maximum stress peak is formed at the upper pressure sensor 5. The greater the grouting pressure, the higher the maximum stress peak. When the slurry exceeds the position of the pressure sensor, the flowing slurry produces upward extrusion pressure on the accumulated rock above the pressure sensor, which causes the pressure on the pressure sensor to weaken and begin to enter the pressure relief stage, and the stress decreasing trend gradually becomes gentle.
4. At the same diffusion height, the viscosity of gangue slurry with the same concentration increases with the increase of grouting pressure, and the higher the grouting pressure, the greater the change of slurry viscosity. Under different grouting pressures, the viscosity of slurry increases steadily with the increase of diffusion height. When the slurry is diffused to the same height, the viscosity change of the low-concentration slurry is smaller than that of the high-concentration slurry. The difference in viscosity change shows an increasing trend with the higher diffusion height.

## Data Availability

The data are contained within the article.
